# Chemotherapy in Ewing’s sarcoma

**DOI:** 10.4103/0019-5413.69305

**Published:** 2010

**Authors:** Sandeep Jain, Gauri Kapoor

**Affiliations:** Department of Pediatric Hematology and Oncology, Rajiv Gandhi Cancer Institute and Research Centre, Delhi, India

**Keywords:** Ewing’s sarcoma, chemotherapy, metastasis

## Abstract

Ewing’s sarcoma constitutes three per cent of all pediatric malignancies. Ewing’s sarcoma has generally been more responsive to chemotherapy than adult-type sarcomas, and chemotherapy is now recommended for all patients with this disease. It is essential to integrate local control measures in the form of surgery and/or radiotherapy at the appropriate time, along with chemotherapy to eradicate the disease. This approach has improved the survival substantially to the tune of 70% in localized disease, although outcome for metastatic disease remains dismal. Newer therapeutic approaches are required to improve outcome for metastatic and recurrent or refractory Ewing’s sarcoma in organized co-operative group trials.

## INTRODUCTION

Ewing’s sarcoma is the second most common primary malignant bone tumor after osteosarcoma, accounting for three per cent of all childhood malignancies.^1^ Initially, it was treated with radiation or surgery alone with high fatality rate. In the last three decades, with the addition of systemic therapy in the form of chemotherapy and progress made in multidisciplinary approach, the prognosis of Ewing’s sarcoma has steadily improved.^1^ Metastatic disease at the time of presentation unquestionably remains the most important prognostic factor affecting outcome. Multimodality treatment has resulted in remarkable improvement in survival of patients with localized disease; however, outcome of patients with metastatic or recurrent disease remains dismal.^2^ The newer agents, in combination with conventional chemotherapy, need to be tested in the forthcoming clinical trials to improve the outcome in patients with metastatic disease and to reduce the therapy related long term sequelae in others. Here, we focus on the evolution of chemotherapy in Ewing’s sarcoma, current therapeutic strategy and targeted therapeutic agents that may be used in the near future.

## STAGING AND PROGNOSTIC FACTORS

There is no universally accepted staging available for Ewing’s sarcoma at this time. The American Joint Committee on Cancer (AJCC) suggests that primary bone or extra skeletal Ewing’s sarcoma may be included with their respective bone or soft-tissue sarcoma (STS) staging systems.^3^ Although the AJCC staging includes metastatic disease and tumor size greater or less than 8 cm; nodal status and grade are irrelevant for Ewing’s sarcoma because this cancer rarely spreads to the lymph nodes and, by definition, is high grade.^3^ TNM staging is not clearly established in Ewing’s sarcoma and most of the centers use presence or absence of metastasis at diagnosis as the main tool to plan treatment strategy.^3^

Ladenstein *et al*.^4^ presented a study on prognostic scoring at diagnosis in prospectively treated patients with primary extra-pulmonary metastatic Ewing’s tumors. Various prognostic factors in that study were incorporated in the proposed TNM staging by the same authors in International Society of Pediatric Oncology SIOP 2009, based on analysis of 1799 Ewing’s sarcoma patients.^5^ Tumor size (> 500 ml), presence of lymph node and distant metastasis were statistically significant prognostic factors with *P* values 0.0001 each. This staging may be used as a basis for stratifying patients to intensify treatment for high risk patients in the future trials.

Various studies have found the following prognostic factors to affect outcome in Ewing’s sarcoma and these include tumor site and size at presentation, age and gender of the patients, serum lactate dehydrogenase (LDH), presence or absence of metastatic disease and response to chemotherapy.^1^,^2^,^6^ Patients with Ewing’s sarcoma of the distal extremity have the best prognosis; those involving the proximal extremity have an intermediate prognosis while those with central or pelvic sites have the worst.^1^,^2^,^6^ Tumor volume has been shown to be an important prognostic factor in most studies. Cutoffs of either 100 ml or 200 ml are used to define larger tumors.^1^ Infants and younger patients (< 15 years) have a better prognosis than adolescents aged 15 years or older or adults.^1^,^2^,^6^ Girls with Ewing’s sarcoma have a better prognosis than boys.^6^ Raised serum LDH levels prior to treatment are associated with inferior prognosis and also correlate with large primary tumors and presence of metastatic disease.^6^ The presence or absence of metastatic disease is the single most powerful predictor of outcome. Patients with metastatic disease confined to lung have a better prognosis than patients with extra pulmonary metastatic sites,^1^,^2^ while patients with metastasis to bone only seem to have a better outcome than patients with metastases to both bone and lung.^7^ Patients with minimal or no residual viable tumor after presurgical chemotherapy have a significantly better event-free survival compared with patients with larger amounts of viable tumor.^8^,^9^

## EVOLUTION OF CHEMOTHERAPY

The past 30 years have witnessed great improvements in the outcome of patients with Ewing’s sarcoma, largely through multidisciplinary approaches tested in cooperative trials. The use of adjuvant chemotherapy, which began in the early 1970s, resulted in a marked improvement in the outcome. Before the era of chemotherapy, the survival of children with Ewing’s sarcoma was only 10%, despite the well known radio sensitivity of this tumor.^10^,^11^ Most patients succumbed to distant relapse, thus necessitating the need for systemic chemotherapy. The use of chemotherapy in Ewing’s sarcoma was first reported in the early 1960s.^12^–^14^ In 1962, Sutow and Sullivan^12^ independently published the use of cyclophosphamide in Ewing’s sarcoma. Subsequently, Hustu *et al*.^14^ published the use of vincristine and cyclophosphamide along with radiotherapy in five patients resulting in sustained complete remission. In 1974, Rosen *et al*.^15^ from Memorial Sloan-Kettering Cancer Center used these agents combining with actinomycin D and doxorubicin (VACD), which marked the beginning of the era of multimodality therapy.

Most of the advances in chemotherapy have come as a result of multicentric trials which have now become the standard of care for evaluating treatment options in Ewing’s sarcoma. For the sake of simplicity, various clinical trials may be grouped in three categories.

The first group of trials established a clear benefit in terms of survival using VACD in different combinations compared to the historical groups. The first Intergroup Ewing’s Sarcoma Study (IESS) (1973 - 1978) was a pioneering collaborative study by Nesbit who brought together the different children’s cancer study groups in the United States to undertake clinical trials in Ewing’s sarcoma. It showed a five-year disease survival of 60% with addition of doxorubicin compared to 24% with vincristine, actinomycin D, cyclophosphamide VAC alone. This trial established unequivocally, the survival advantage with regimes using doxorubicin in addition to VAC.^16^ It was also demonstrated that inclusion of doxorubicin with every cycle is superior to the use of doxorubicin alternate with actinomycin D, even when the cumulative doses of both the drugs in two schedules were identical. The addition of prophylactic whole lung radiotherapy improved outcomes, although not as much as addition of doxorubicin.

The IESS-II trial (1978-1982) demonstrated that intermittent high dose therapy with VAC plus doxorubicin (150% increase in the initial weeks of therapy) was superior to continuous moderate dose therapy with these agents.^17^ This highlighted the importance of increasing doxorubicin intensity early in the course of therapy and aggressive cytoreduction. Since then, many multi-institutional collaborative trials both within (IESS- I,^16^ IESS- II,^17^ ES-79,^18^ and Pediatric Oncology Group POG 8346 19 and outside the United States (Germany’s CESS 81,^20^ UK’s ET-1,^21^) have confirmed the clinical benefit of VACD-based regime.

The second group of trials aimed at improving the survival of patients by incorporating either etoposide or ifosfamide or both to preexisting regimes. Ifosfamide and etoposide (IE) have been found to be very effective against this tumor as these agents have a synergistic antitumor effect and the efficacy of both agents improve with fractionated administration. Craft and coworkers reported an improvement in five-year survival from 44% as published in Ewing’s Tumor (ET)-1 study to 62% in ET-2 study using vincristine, actinomycin-D, doxorubicin and ifosfamide compared with VAC plus doxorubicin.^22^ The first American Intergroup Ewing’s trial (INT-0091 - POG-8850/CCG-7881) evaluated the use of IE in front line treatment of Ewing’s sarcoma family of tumors (ESFT) and all patients were randomized to receive VACD with or without ifosfamide, etoposide (IE).^23^ The addition of IE did not prove to be advantageous for patients with metastatic disease; with a five-year event-free survival (EFS) of 22% for both the experimental and standard arms probably underscoring the inherent biologic differences of this subgroup of patients. On the other hand, the VACD/IE regimen was superior to the standard VACD (five-year EFS 69% *versus* 54% respectively, *P* = 0.005) for patients with localized disease. The greatest beneficial effect of the incorporation of the IE pair was for patients with large tumors and patients with pelvic primaries. This study also demonstrated that the benefit of more intensive chemotherapy was not limited to its systemic effects, but was also advantageous for local control.

The third group of trials aimed at improving the survival by dose dense therapies. Because of high chemo sensitivity of Ewing’s sarcoma and steep dose response curve of alkylating agents, dose intensification to improve survival has been of much interest. Use of growth factors has made it feasible to intensify the treatment without increasing the treatment related morbidities.^24^ The importance of dose intensification in the treatment of Ewing’s sarcoma has also been evaluated in the second American Intergroup POG-CCG Ewing’s trial (POG-9354/CCG-7942), in which patients were randomized to receive the treatment protocol for either 30 or 48 weeks. The cumulative doses of agents were similar in both arms, but in the 30-week arm, higher doses per cycle were given. The five-year EFS and overall survival rates for all eligible patients were 71.1% (95% CI, 67.7 to 75.0%) and 78.6% (95% CI, 74.6 to 82.1%), respectively. There was no significant difference (*P*=0.57) in EFS between patients treated with the standard (five-year EFS, 72.1%; 95% CI, 65.8 to 77.5%) or intensified regimen (five-year EFS, 70.1%; 63.9 to 75%). Thus, dose escalation of alkylating agents as tested in this trial did not improve the outcome for patients with nonmetastatic ESFT of bone or soft tissue.^26^ An alternative to increasing dose intensity is decreasing the interval between cycles while maintaining the same dose-per-cycle with the use of G-CSF. In the US, this is the approach taken by the Children’s Oncology Group AEWS-0031 study, in which, patients with non-metastatic extradural ESFT were randomized to receive alternating cycles VDC and IE every three weeks (standard arm) or two weeks (dose-compression arm), resulting in 33% dose intensification. This dose intensification and interval compression has the theoretical advantage of allowing less time for recovery of partially resistant cells. The three-year EFS of the two groups were 65% versus 76% (*P*=0.028) respectively for standard versus dose-compression arm, without increased toxicity in the latter.^27^ This is currently the standard of care for localized disease in COG studies.

## CURRENT STANDARD OF CARE

It is well established that chemotherapy is the mainstay of treatment in Ewing’s sarcoma and is a necessary addition to local control in order to achieve a reasonable expectation of cure.^12^–^16^ The treatment plan generally consists of three stages: initial cytoreduction with chemotherapy to eradicate micro metastatic disease and facilitate effective local control measures with wide negative margins; definitive radiation or surgical therapy to eradicate all known disease; and consolidation therapy for eradication of occult residual disease to reduce the likelihood of tumor recurrence. Importantly, neoadjuvant chemotherapy not only helps achieve optimal cytoreduction to facilitate limb salvage procedures but also provides a chance to assess the response to chemotherapy.^8^,^9^

### Non metastatic disease

Till recently, protocols for non-metastatic disease in the US generally consisted of POG 9354, which includes alternate courses of VDC with courses of IE every three weeks^26^ for 48 weeks with local control at 9-12 weeks. However, after publication of the results of Children’s Oncology Group AEWS-0031 study, current standard of care is administration of similar agents every two weekly.^27^ Treatment results of localized Ewing’s sarcoma are listed in Table 1.^15^–^18^,^20^–^23^,^25^–^32^ European EURO EWING 99 trial combines vincristine, doxorubicin, ifosfamide and etoposide in a single treatment cycle for 42 weeks with local control at week 15.^33^

**Table 1:** Treatment results in selected clinical studies of localized Ewing’s sarcoma

Study	Reference	Schedule	Patients	5-year EFS*	*P* value	Comments
IESS studies						
IESS-I (1973-1978)	Nesbit *et al*.^16^	VAC	342	24	VAC vs. VAC+WLI, .001	Value of D
		VAC+WLI		44	VAC vs. VACD, .001	Benefits of WLI?
		VACD		60	VAC +WLI vs.VACD, .05	
IESS-II (1978-1982)	Burgert *et al*.^17^	VACD-HD	214	68	.03	Value of aggressive cytoreduction
		VACD-MD		48		
First POG-CCG INT-0091 (1988-1993)	Grier *et al*.^23^	VACD	200	54	.005	Value of combination IE in localized disease, no benefit in metastatic disease.
		VCAD+IE	198	69		
Second POG- CCG (1995-1998)	Granowetter *et al*.^25^	VCD+IE48 weeks	492	75 (3 yrs)	.57	No benefit of dose time compression
		VCD+IE30 weeks		76 (3yr)		
Memorial Sloan-Kettering Cancer center studies						
T2 (1970-1978)	Rosen *et al*.^15^	VACD (adjuvant)	20	75		After local therapy only, cumulative dose of D upto 600 mg/m^2^
P6 (1990-1995)	Kushner *et al*.^28^	HD-CVD+IE	36	77 (2yr)		C dose escalation 4.2 g/m^2^ per course
P6 (1991-2001)	Kolb *et al*.^29^	HD-CVD+IE	68			Good result in localized disease, poor outcome in metastatic patients.
		Localized		81 (4yr)		
		Metastatic		12 (4 yr)		
St. Jude studies						
ES-79 (1978-1986)	Hayes *et al*.^18^	VACD	52			Tumor size as prognostic factor
		<8cm		82 (3yr)		
		>8cm		64 (3yr)		
ES-87 (1987-1991)	Meyer *et al*.^30^	Therapeutic window with IE	26			Combination IE effective
			Clinical response	96		
EW-92 (1992-1996)	Marina *et al*.^31^	VCD-IEx3	34	78 (3yr)		Tumor size (<or>8cm) loses prognostic relevance with more intensive treatment
		VCD/IE intensified				
UKCCSG/MRC studies						
ET-1 (1972-1986)	Craft *et al*.^21^	VACD	120	41		Tumor site as the most important prognostic factor
		Extremity		52		
		Axial		38		
		Pelvic		13		
ET-2 (1987-1993)	Craft *et al*.^22^	VAID	201	62		Importance of the administration of high-dose alkylating agents(I)
		Extremity		73		
		Axial		55		
		Pelvic		41		
CESS studies						
CESS-81 (1981-1985)	Jurgens *et al*.^20^	VACD	93			Tumor volume (<or>100ml) and histological response are
		<100ml		80		
		>100ml		31 (3yr)		
CESS-86 (1986-1991)	Paulussen *et al*.^32^	(SR)VACD <100ml	301	52 (10yr)		Intensive treatment with I for high risk patients. Tumor volume (<or>200ml) and histologic response as prognostic factor
		(HR)VAID >100ml	51 (10yrs)			

*P* values are given only for trials comparing randomized treatment arms.

* Values are in percentages. Abbreviations: A: Actinomycin D, C: Cyclophosphamide, CESS: Cooperative Ewing’s sarcoma studies, D: Doxorubicin, E: Etoposide, EFS: Event –free survival, EICESS: European Intergroup Cooperative Ewing’s Sarcoma, HD: High dose, HR: High risk, I: Ifosphamide, IESS: Intergroup Ewing’s Sarcoma Study, MD: Moderate dose, MRC: Medical Research Council, NA: Not available, P cisplatinum, POG-CCG: Pediatric Oncology Group-children’s Cancer Group, ROI: Rizzoli Orthopedic Institute, SFOP: French Society of Pediatric Oncology, SSG: Scandinavian Sarcoma Group, SR: Standard risk, UKCCSG: United Kingdom Children’s Cancer Study Group, V: Vincristine, WLI: Whole lung irradiation

### Metastatic disease

Ewing’s sarcoma has a potential for hematogenous metastasis and the most common sites of metastases include lungs, bones and bone marrow. About 25% of patients have metastatic disease at presentation and their overall survival is dismal.^2^ Moreover, patients with isolated lung metastasis fare better than those with extrapulmonary disease as shown by Cotterill *et al*. who, reported five-year RFS of 29% for those with lung-exclusive metastases, 19% for bone metastases, and 8% for those with combined lung and bone metastases (*P*=0.001).^2^ The chemotherapy regimen and initial treatment for patients with metastatic disease is the same as that for localized disease. At the time of local therapy (after four to six cycles of neoadjuvant chemotherapy), all sites of the disease must be re-evaluated. If tumor shows progression or there is persistence of widespread disease, there is little hope for cure and most such children should be treated with palliative intent. For patients responding well, at this stage, local therapy in the form of surgery and or radiation is recommended to the primary site as well as all metastatic sites. Subsequently, consolidation chemotherapy is continued with similar agents for 30-48 weeks, as determined by the protocol.^26^

### Role of autologous stem cell transplant

Intensification of induction chemotherapy either by dose escalation or the addition of newer agents or consolidation after first complete remission with mega therapy and hematopoietic stem cell rescue has not improved the overall survival significantly.^34^ In general, most conditioning regimens use alkylating agents like thiotepa, busulfan and melphalan.^35^ Kushner *et al*. demonstrated that the overall survival at five years was 44% for the group of 18 patients that received busulfan, and only 23% for the group of 93 patients that were treated with regimens without busulfan. The use of busulfan provided a survival advantage also for patients with pulmonary disease alone (66% versus 39%) and for patients with localized high-risk disease (75% versus 38%).^36^

The lack of benefit of mega therapy in metastatic disease emphasizes the difference in biology of these tumors and the need for novel strategies to deal with them. However, many prospective trials are in process to address this issue and we may get an answer in the near future. Using the principle of graft versus tumor (GVT) effect, certain institutions have reported successful results with allogeneic transplant as well. These results are preliminary and the number of patients is quite small.^37^

### Local therapy for primary tumor site

Although Ewing’s sarcoma is very radiosensitive, this modality is used less frequently now, given the potential morbidities of this approach (secondary malignancies and adverse effects on bone growth). Moreover, because of the advances in surgical techniques that facilitate limb salvage, it is the preferred modality for local control when wide resection margin is possible. Some studies have reported reduced local failure rates (10 or less vs. 30%) with use of surgery when compared to radiotherapy for local control.^38^,^39^ These results should be interpreted with caution as these are retrospective studies susceptible to bias favoring surgical resection of smaller, more peripheral tumors and so far, no prospective data is available. Radiation is, however, recommended for tumors not amenable to surgery or in those resected with compromised margins. Role of postoperative radiotherapy in patients with poor histological response to chemotherapy is controversial. Patients with poor response to presurgical chemotherapy have an increased risk for local recurrence.^38^ However, addition of postoperative radiotherapy as local control measure after wide resection did not translate into overall survival benefit.^38^ Unlike in osteosarcoma, where presence of pathologic fracture independently predicts worse survival, in Ewing’s sarcoma, this is not always so.^40^ Moreover, if the fracture heals with neoadjuvant chemotherapy, its presence does not preclude surgical resection. Radiotherapy is clearly preferred in cases with persistent pathologic fracture after neoadjuvant chemotherapy.

### Local therapy for metastatic sites

The role of radiotherapy to sites of initial metastases and its timing is also debatable. Bilateral pulmonary irradiation at a dose of 14–20 Gy has been reported to improve the outcome of patients with pulmonary disease.^41^,^42^ The EURO-EWING-INTERGROUP-EE99 (COG-AEWS0331) are doing a randomized study for patients with pulmonary metastases only and evaluating standard chemotherapy and peripheral blood stem cell transplant versus standard chemotherapy and bilateral lung radiation. The results of this study may be useful to decide the treatment for patients with lung metastasis, as this is the first randomized study of high dose therapy (HDT) among high risk patients.

### Relapse

Unfortunately, Ewing’s sarcoma is a disease in which relapses are known to occur even several years after completion of treatment. With the advances in local therapy, most of the relapses are either systemic or combined. However, generally prognosis for patients with recurrent disease remains very poor and in contrast to osteosarcoma, patients with recurrent tumors require additional chemotherapy in order to achieve long-term survival. Leavey *et al*. showed that patients who develop recurrent disease within the first two years are even worse with five-year survival around 7% (compared to 30% for recurrent disease after two years).^43^ As discussed earlier, patients with isolated lung relapse fare better than those with bone or bone marrow disease.^44^ In view of lack of any prospective data, there is no standard definition of best treatment for relapsed patients. Of the newer chemotherapeutic agents investigated in Ewing’s sarcoma, the camptothecan derivatives have been the most promising. The topoisomerase I inhibitors, topotecan and irinotecan, have shown efficacy as single agents in many pediatric solid tumors. However, in Ewing’s sarcoma, these agents have not found been to be effective as single agents, although, they have shown promising results in combination with cyclophosphamide. Saylors *et al*. and Bernstein *et al*. showed that the combination of the two has response rate of 36% in recurrent disease and 56% in untreated metastatic disease.^45^,^46^ As part of a clinical trial, topotecan is currently being included in the COG study and is being contemplated for inclusion in upcoming EURO- EWING’s trials. Similarly, the combination of temozolomide and irinotecan has also proved to be effective for Ewing’s sarcoma.^47^–^49^ These results have been confirmed internationally and, at present, either of the two combinations may be considered for use as second-line or salvage therapy. The role of gemcitabine/ taxotere for treatment of Ewing’s sarcoma, remains to be determined by an ongoing SARC (Sarcoma Alliance for Research through Collaboration) sponsored trial.^50^ To conclude, treatment of this subset of patients requires a fresh approach, wherein, international cooperative studies are needed to conceive newer strategies in order to give this last group of patients a fair chance of cure.

## LATE EFFECTS

Owing to the increased number of long-term survivors, late side effects of treatment are more evident and have been better studied.^51^–^53^ Late effects in Ewing’s sarcoma may be attributed to local therapy i.e. either surgery or radiation or systemic chemotherapy. Surgery may lead to suboptimal functional outcome of the limb, resulting in impaired body image.^54^ Limb salvage procedures may be complicated with prosthesis infection and delayed amputation.^54^ Radiotherapy may be associated with growth disturbances, musculoskeletal abnormalities and development of second malignancy.^51^

Therapy-related second malignancy is the most devastating complication after successful treatment of primary cancer. The cumulative incidence of second neoplasm in most large series is lower than two per cent.^51^ The incidence of secondary leukemia is protocol driven and high dose chemotherapy is associated with increased incidence and occurs usually within three years of initial diagnosis.^51^ Etoposide is an associated risk factor for second malignancy and its exposure has been linked to the occurrence of a second malignancy in the regimens that implicated high-dose therapy even more strongly.^51^ The IESS trial that compared VDC with VDC-IE showed no difference in second malignancies between therapeutic arms, suggesting that in the dose and schedule employed, the addition of etoposide did not independently increase the risk for a second malignancy.^23^ On the other hand, it is notable that C-arm of the Children’s Cancer Group– Pediatric Oncology Group Intergroup study INT 0091, designed for metastatic Ewing’s, in which very high cumulative doses of ifosfamide (140 g/m^2^) and cyclophosphamide (17.6 g/m^2^) were prescribed, demonstrated 10% incidence of therapy-related leukemia.^23^

Other complications of chemotherapy are agent specific. Anthracyclines, including doxorubicin are known to cause chronic cardiomyopathy in a dose related manner.^52^ Steinherz *et al*. reported an incidence of 23% echocardiographic abnormalities with median cumulative dose of 450 mg / m^2^ at seven years.^52^ Thus, cumulative dose of doxorubicin is usually limited to less than 450 mg/m^2^. In addition, either prolonged administration or administration of dexrazoxane prior to doxorubicin may reduce the toxic effects of doxorubicin on the myocardium.^53^ The alkylating agents like cyclophosphamide and ifosfamide are associated with infertility, especially male infertility, so that sperm cryopreservation should be offered to post pubertal boys prior to the administration of chemotherapy. In addition, ifosfamide can cause a persistent renal tubular electrolyte loss and, less commonly, a decrease in glomerular function, again in a dose-dependent fashion.

## FUTURE TRENDS

With the use of multimodality therapy, the survival of localized Ewing’s sarcoma has improved considerably, however, outcome of a sub group of patients with metastatic disease or recurrent disease remains dismal.^2^,^43^ Poor outcome of these patients highlights the need for novel chemotherapeutic agents and targeted therapy.

EWS-FLI fusion protein is unique and is present in 85% of Ewing’s sarcoma. Targeted therapy against this fusion protein or its products may inhibit growth of Ewing’s sarcoma cells.^55^ However, efficient delivery of EWS-FLI antisense oligonucleotide to malignant cells remains a barrier to therapeutic application of these agents. Nanocapsules and nanospheres have been tried in animal studies but its application in humans is still evolving.^56^

The role of insulin like growth factor-1 (IGF-1) and its receptor IGF-1R in the pathogenesis of Ewing’s sarcoma is well established.^57^ IGF-1R is found on the surface of most Ewing’s sarcoma cells and is necessary for the transforming ability of EWS-FLI fusion proteins.^57^ Thus, targeted therapy against IGF-1/ IGF-1R may be a very effective strategy in future. In animal trials, IGF and IGF-1R target therapy have been found to be effective to reduce the tumoorigenic and metastatic ability of Ewing’s sarcoma cells and improve the efficacy of conventional chemotherapy.^58^ IGF-1R targeted therapy results in synergistic effects with doxorubicin and vincristine due to the induction of apoptosis which has additional clinical implication.^59^ Several phase I trials, including one conducted at M.D. Anderson using R1507 (a humanized monoclonal anti-IGF-1R antibody developed by Roche, Nutley, New Jersey, USA), suggest a high degree of safety with no dose-limiting toxicities observed at the highest dose level evaluated (presented in abstract form at the 2007 American Association for Cancer Research Molecular Targets — National Cancer Institute- European Organization for Research and Treatment of Cancer International Conference). Although not designed for clinical efficacy, two of the seven Ewing’s sarcoma family tumor (ESFT) patients enrolled in the phase I trial had near complete responses, suggesting this strategy can benefit at least a subset of ESFT patients.^60^ Clinical applications of these molecules are in experimental stages. Cell surface transmembrane protein CD99 encoded by the *MIC2* gene is consistently expressed in Ewing’s sarcoma cell lines. Although neither its function nor its ligand has been identified but its role to induce massive apoptosis through caspase-independent mechanisms has been studied in mice.^59^ Systemic delivery of the anti-CD99 antibodies significantly reduced the number of lung and bone metastases.^59^ Thus, it seems that the combination of anti-CD99 monoclonal antibodies with conventional chemotherapeutic agents may be a useful strategy in the future. Clinical trials using anti-CD99 antibodies have not yet been attempted because of high levels of CD99 expression in hematopoietic cell line, pancreas, and gonads.

Rapamycin, is a highly specific inhibitor of mTOR, a serine/threonine kinase that controls cap-dependent translation. Inhibition of mTOR signaling potentially inhibits cell cycle regulators as well as transcription factors such as c-Myc. mTOR inhibitor causes inhibition of Ewing’s sarcoma cells *in vitro*, suggesting a possible therapeutic role in ESFT.^61^

## INDIAN SCENARIO

There is paucity of data on Ewing’s sarcoma from India, owing to the lack of uniform policy of cancer reporting and maintenance of cancer registry. Iyer *et al*. from Tata Memorial Hospital, Mumbai reported a 55% disease-free survival at three years in 28 patients with localized Ewing’s sarcoma using vincristine, cyclophosphamide, and adriamycin.^62^ The same group later published results on 50 patients, five of whom had metastatic disease with disease-free survival of 38.0% ± 2.5% at five years.^62^,^63^ At our Institute, we follow POG 9354 protocol wherein, we administer four to five cycles of neoadjuvant chemotherapy vincristine, adriamycin and cyclophosphamide alternate with etoposide and ifosfamide before local therapy that includes surgery and/or radiotherapy followed by continuation chemotherapy with similar agents, to complete a duration of 48 weeks.

## CONCLUSION

Ewing’s sarcoma is essentially a systemic disease with clinically evident or micro metastatic disease at presentation. Thus, chemotherapy remains the backbone of treatment with multimodality approach. Local therapy with surgery and/or radiotherapy is an important component of therapy. With the advent of effective systemic chemotherapy, the prognosis of patients with localized Ewing’s sarcoma has improved remarkably. However, durability of response in metastatic or recurrent disease remains elusive. It is hoped that research in tumor biology will provide a better understanding of pathogenesis and progression of disease, thereby providing novel therapeutic agents that may provide safe and effective treatment of these patients.
